# Association of Fried Food Intake with Gastric Cancer Risk: A Systemic Review and Meta-Analysis of Case–Control Studies

**DOI:** 10.3390/nu15132982

**Published:** 2023-06-30

**Authors:** Ting Zhang, Sang Shin Song, Meiling Liu, Sunmin Park

**Affiliations:** 1 Department of Bioconvergence, Hoseo University, Asan 31499, Republic of Korea; zhangting92925@gmail.com; 2 Department of Food and Nutrition, Obesity/Diabetes Research Center, Hoseo University, Asan 31499, Republic of Korea; ssscho@empas.com; 3 Department of Chemical Engineering, Shanxi Institute of Science and Technology, Jincheng 048011, China; meiling1125@naver.com

**Keywords:** fried foods, cooking methods, gastric cancer, meta-analysis, Asians, non-Asians

## Abstract

Gastric cancer is one of the most prevalent cancers in Asia, and has a significant global incidence. However, the impact of fried food consumption on gastric cancer risk remains uncertain, mainly due to the limited number of participants in previous studies. To address this knowledge gap, we aimed to examine the association between fried food intake and gastric cancer incidence through a comprehensive meta-analysis. We conducted a thorough search across multiple databases, including PubMed, EMBASE, Google Scholar, Cochrane Library, China National Knowledge Infrastructure (CNKI), Korean Information Service System (KISS), and Research Information Service System (RISS), to collect studies. The newly analyzed results of the Korean Genome and Epidemiology Study (KoGES) findings were added. We assessed integrated odds ratios (ORs) and their corresponding 95% confidence intervals (CIs) from the selected studies using Cochrane RevMan 5.0 for the meta-analysis. The quality of the studies included in the meta-analysis was assessed using the Study Quality Assessment Tool of the National Heart, Lung, and Blood Institute (NHLBI). We included 18 studies in the analysis, which compared the impact of fried food intake in gastric cancer patients (*n* = 5739) and healthy adults (control, *n* = 70,933). There was a significant positive association between gastric cancer risk and fried food intake (OR = 1.52, 95% CI = 1.23–1.87, I^2^ = 76%, *p* = 0.0001). The relationship was found to be significant in both non-East Asians (OR = 1.48, 95% CI = 1.18–1.85, I^2^ = 31%, *p* = 0.0006) and East Asians (OR = 1.54, 95% CI = 1.14–2.08, I^2^ = 83%, *p* = 0.005). In conclusion, this meta-analysis supports the notion that fried food intake is associated with an increased risk of gastric cancer in both non-Asians and Asians. Promoting a reduction in fried food consumption as a measure against gastric cancer risk is recommended.

## 1. Introduction

According to the 2020 estimates by the International Agency for Research on Cancer (IARC), the specialized cancer agency of the World Health Organization (WHO), gastric cancer (5.6%) is ranked as the fifth most common cancer worldwide, with a mortality rate (7.7%) that places it fourth among cancer-related deaths [1]. Although gastric cancer’s incidence has declined globally, it remains notably high in Korea compared to other regions, consistently ranking as the second-most prevalent cancer, after thyroid cancer, until 2018 [2].

The relationship between dietary factors and gastric cancer risk has been extensively investigated. In 2018, the World Cancer Research Fund (WCRF) and the American Institute for Cancer Research (AICR) reported that there was no convincing evidence linking dietary factors to gastric cancer risk. However, they identified certain potential risk factors [3]. WCRF/AICR have reported that there is probable evidence that increased intake of alcohol and salted foods increases the risk of gastric cancer. There is some evidence that consuming grilled or barbecued meat and fish, processed meat, and little or no fruit increases the gastric cancer risk, while citrus fruit decreases it. Furthermore, several risk factors have been implicated in gastric cancer, including *Helicobacter pylori* (*H. pylori*) infection, a diet high in salt, the consumption of smoked or pickled foods, tobacco use, obesity, and a family history of the disease [3]. The WCRF/AICR recommendations emphasize the importance of limiting the intake of fast and processed foods high in fat, starches, and sugars for cancer prevention [3].

The specific role of high-fat foods, particularly fried foods, in gastric cancer risk remains uncertain. During the frying process, the chemical composition of food components and oils undergoes changes through polymerization, hydrogenation, and oxidation, which can lead to the formation of potentially carcinogenic substances such as acrylamide and heterocyclic amines [4]. Case–control studies have demonstrated the carcinogenic role of fried foods in several cancers, including the incidence of pharynx [5], larynx [6], gallbladder [7], and prostate cancers [8]. However, other studies have shown either no or an inverse association between fried foods and cancers [9]. Despite the potential evidence indicating a positive association between fried food consumption and cancer risk, it is noteworthy that there has been a noticeable rise in the number of fast-food restaurants, accompanied by increased demand for fried foods.

We conducted a comprehensive meta-analysis to address the discrepancies in the existing literature regarding the association between fried food intake and gastric cancer risk. Our meta-analysis encompassed 17 published studies, and we added the newly analyzed findings from the Korean Genome and Epidemiology Study (KoGES) to examine the relationship between fried food intake and gastric cancer risk. By combining these studies, our objective was to assess the overall association between fried food intake and gastric cancer risk among both non-East Asians and East Asians. In this study, we present the findings of our meta-analysis, which provides significant evidence supporting a positive association between fried food intake and the risk of gastric cancer in both non-East Asians and East Asians. These results contribute to our understanding of the potential impact of fried foods on gastric cancer risk and highlight the importance of reducing fried food consumption.

## 2. Methods

### 2.1. Data Sources and Literature Selection Criteria

A systematic review and meta-analysis were conducted to evaluate the association between fried food intake and gastric cancer risk. The study followed the Preferred Reporting Items for Systematic Reviews and Meta-Analyses (PRISMA) guidelines to ensure transparent reporting and rigorous methodology. The meta-analysis included epidemiological studies published between 1990 and 2022. All of the above studies were limited to cohort or case–control designs published in the English, Chinese, and Korean languages. The English databases used were EMBASE, PUBMED, Google Scholar, and the Cochrane Library; the Chinese database was the China National Knowledge Infrastructure (CNKI); and the Korean databases were the Research Information Service System (RISS) and the Korean Information Service System (KISS). The literature search was conducted using the following keywords: fried foods, dietary habits, cooking methods, and gastric cancer.

### 2.2. Literature Evaluation and Selection

To evaluate the association between fried food intake and gastric cancer risk, we analyzed the total number of gastric cancer patients and controls (persons without cancer) in the selected studies among the high- and low-fried-food-intake groups. Studies that defined gastrointestinal cancer as gastric cancer or included smoked, grilled, or processed meat foods in the category of fried foods were excluded.

### 2.3. Risk of Bias Assessment of the Research Literature

We evaluated the quality of the case–control studies in the analysis using the Study Quality Assessment Tool of the National Heart, Lung, and Blood Institute (NHLBI) [10]. This tool included 13 categories of assessment, as follows: (1) research question; (2) study population; (3) target population and case representation; (4) sample size justification; (5) groups recruited from the same population; (6) inclusion and exclusion criteria, prespecified and applied uniformly; (7) case and control definitions; (8) random selection of study participants; (9) concurrent controls; (10) exposure assessed prior to outcome measurement; (11) exposure measures and assessment; (12) blinding of exposure assessors; and (13) statistical analysis. The answer to each question was assigned a score, as follows: yes (0); NA, i.e., not applicable or not mentioned (1); or no (2).

Two independent reviewers (S.P. and T.Z.) evaluated the quality of the studies. If there was a disagreement among the reviewers regarding the scores, they resolved it through discussion. This rigorous evaluation process ensured the high quality and reliability of the meta-analysis.

### 2.4. Data Extraction

The following information was collected from each study eligible for our analysis: first author’s surname, publication year, country, study period, gender of participants, number of cases and controls, types of gastric cancer, comparison of exposure level to fried foods (high versus low), multivariate-adjusted OR with corresponding 95% CI for the high and low categories of fried food intake, and covariates adjusted in the statistical analysis. The maximally adjusted model was selected when a study provided gastric cancer risk estimates with various covariates. Fried food intake was divided into two levels, high and low, and the categories of intake levels were defined in each study. Among the 18 case–control studies, 6 studies used crude data [11,12,13,14,15,16] and 12 studies used adjusted data with covariates [17,18,19,20,21,22,23,24,25,26,27,28]. Thirteen studies showed a significant increase in risk [11,12,13,14,16,18,20,22,24,27,28], two studies showed a significant decrease [15,17,21], and the other three studies reported an unclear risk [19,23,25,26].

The low- and high-fried-food-intake groups were categorized based on the cutoff for fried food intake in each study. Although the fried food intake cutoff was somewhat different in each study, it was generally less than once a week; this was considered the low intake group.

### 2.5. Statistical Analysis

The Cochrane RevMan v 5.0 was used for all statistical analyses [29]. We reported the odds ratios (ORs) and 95% confidence intervals (CIs) from each study, comparing the high and low categories of fried food intake. Heterogeneity between the studies was estimated using the I^2^ statistic, and the I^2^ values ranged from 0% to 100%, with 0% being scored when no heterogeneity was observed between studies. Pooled ORs were applied to the forest using either a fixed-effects model (used in the absence of heterogeneity, I^2^ < 50%) or a random-effect model (used in the presence of heterogeneity, I^2^ > 50%). Statistical significance was determined at the significance level of *p* < 0.05 [30]. To explore the potential heterogeneity among studies, we conducted subgroup analyses for population regions (East Asian and non-East Asian). We visually inspected the funnel plot symmetry and performed the Egger linear regression test to assess the possibility of a publication bias [31]. Asymmetric and symmetric funnel plots suggest a high and low risk of study bias, respectively.

## 3. Results

### 3.1. Study Selection and Study Characteristics

**Figure 1:**
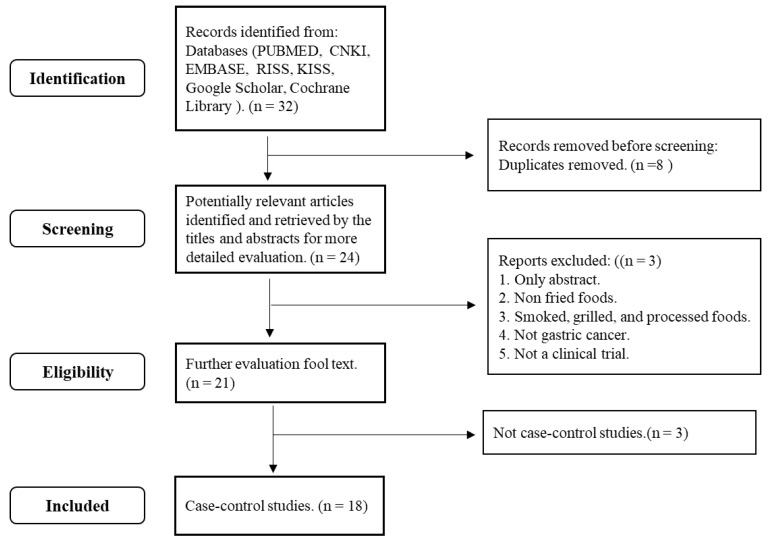
Flowchart of the case–control study selection process related to fried foods and gastric cancer.

Figure 1 outlines the selection process of the publications on the association between gastric cancer and fried food intake. A total of 32 studies were selected in the initial search with keywords, and 8 duplicates were removed. Among the remaining 24 studies, three studies providing only abstracts; with food content including smoked, grilled, and processed foods; or covering gastrointestinal cancer patients were excluded. A further 3 studies that were not case–control studies were excluded. The result analyzed by our research team using KoGES was added. Finally, the meta-analysis included 18 articles after the above exclusions and one inclusion.

Table 1 shows the main characteristics of the studies included in the analysis. Five population-based case–control studies [15,16,19,21,26] and thirteen hospital-based case–control studies were included [11,12,13,14,17,18,20,22,23,24,25,27,28]. Thirteen studies were published in the 2000s [12,13,14,15,16,21,22,23,24,25,26,27,28], whereas five studies were published in the 1990s [11,17,18,19,20]. The East Asian studies were conducted in China (*n* = 9) [13,15,16,19,20,21,22,24,25] and Korea (*n* = 3) [11,14,28], and the non-East Asian studies were conducted in the Middle East (*n* = 3) [17,26,27], Europe (*n* = 1) [18], and South America (*n* = 2) [12,23].

An association between gastric cancer and fried food intake in the hospital-based cohort in KoGES (*n* = 48,306) has not been evaluated or published. Therefore, the association of gastric incidence with fried food was conducted using logistic regression after adjusting for covariates such as age, gender, education, residence area, body mass index (BMI), and energy intake in KoGES. The total number of gastric cancer patients and normal control subjects without a history of cancer were 5739 and 70,933, respectively.

### 3.2. High Intake of Fried Foods

**Figure 2:**
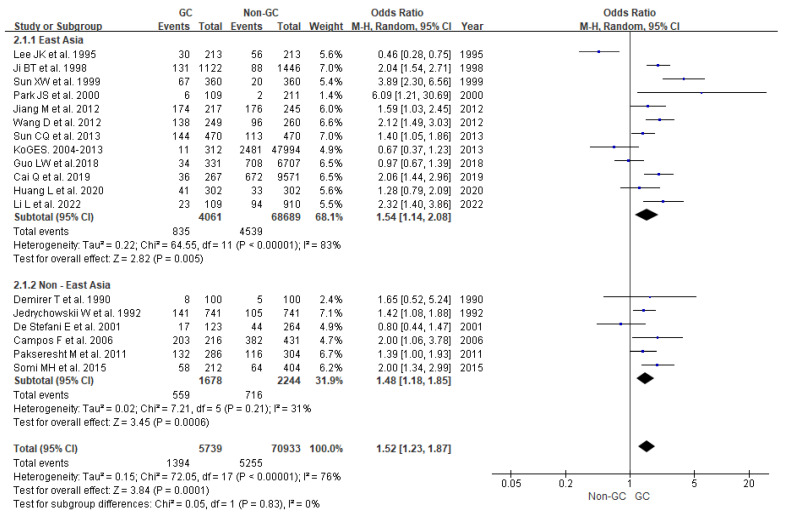
Forest plot of the association between high fried food intake and gastric cancer risk. Data from references [11,12,13,14,15,16,17,18,19,20,21,22,23,24,25,26,27,28].

The OR and 95% CI of the high- versus low-fried-food-intake categories from each study are shown in Table 2. A forest plot of the 18 studies is shown in Figure 2. The pooled results of the 18 studies showed a significant positive association between the risk of gastric cancer and fried food intake (OR = 1.52, 95% CI = 1.23–1.87, I^2^ = 76%, *p* = 0.0001).

We also conducted subgroup analyses based on the participants from different regions (East Asian and non-East Asian). A statistically significant and positive association between the high intake of fried foods and gastric cancer risk was observed among non-East Asians (OR = 1.48, 95% CI = 1.18–1.85, I^2^ = 31%, *p* = 0.0006) and East Asians (OR = 1.54, 95% CI = 1.14–2.08, I^2^ = 83%, *p* = 0.005).

### 3.3. Sensitivity Analysis and Publication Bias

**Figure 3:**
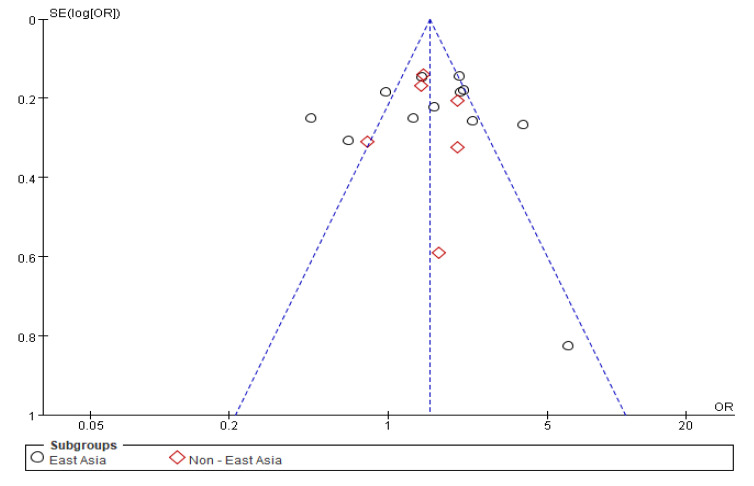
Funnel plot of the meta-analysis assessing the risk of a high fried food intake leading to gastric cancer. Note: dotted lines represent a 95% pseudo-confidence interval. SE: standard error; OR: odds ratio.

A sensitivity analysis was performed to assess the effect of excluding an individual study (t = 0.03, *p* = 0.98), suggesting that no single study had a significant impact. The funnel plot was symmetrical (Figure 3), and the publication bias, according to Egger’s test, was insignificant (*p* = 0.67). These results indicated that there was no publication bias in the selected studies.

### 3.4. Risk of Bias (ROB)

**Figure 4:**
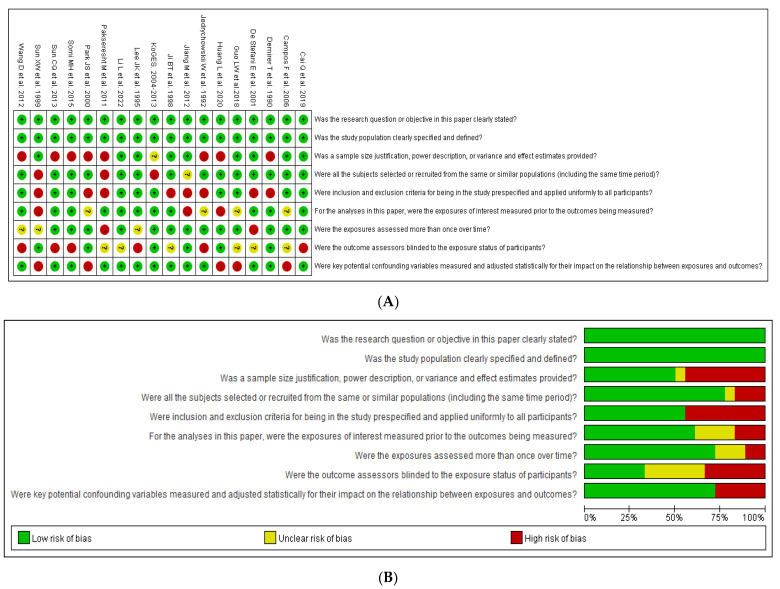
Summary of risk of bias (ROS) of case–control included in the systematic review and meta-analysis. Data from references [11,12,13,14,15,16,17,18,19,20,21,22,23,24,25,26,27,28] (**A**) ROS graph pooling 18 RCTs. (**B**) ROS summary of each RCT. 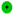 Low risk of bias; 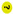 Uncertain risk of bias; 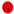 High risk of bias.

The ROB for determining the quality of the included case–control studies is shown in Supplementary Table S1. Some studies included an uncertain or high ROB for some items. The ROB for the included case–control studies was determined, and is illustrated in Supplementary Table S1 and Figure 4. Overall, over 79% of the studies had low ROBs, but some studies had uncertain or high ROBs for specific items. All studies used appropriate randomization methods, such as coin tossing, dice, and random number generation, and most studies were blinded to the participants and personnel. However, some studies did not follow blinding methods for the purpose of allocation concealment and outcome assessment. Incomplete outcome data were detected in some studies, which may have employed selective reporting. Overall, the ROBs of the included studies were low.

## 4. Discussion

Despite a gradual decrease in the worldwide incidence of gastric cancer over the last 50 years, it remains one of the most prevalent cancers in Asia. Dietary intake and cooking methods are believed to play significant roles in gastric cancer development due to the direct contact between food and gastric mucosa. However, the association between fried food consumption and gastric cancer incidence remains controversial. This study presents the results of a meta-analysis of 18 case–control studies of the association between fried food intake and gastric cancer risk. A high level of fried food intake was positively associated with the risk of gastric cancer, and this association was statistically significant in both the non-East Asian and East Asian regions. The pooled results of the 18 studies showed a significant positive association between fried food intake and the risk of gastric cancer.

Several epidemiological studies have already reported a positive association between food cooked at high temperatures (grill, barbecue, frying, etc.) and the risk of colorectal cancer [32,33]. The body converts nitrate to nitrite during metabolic processes, and nitrite has been implicated in the development of various cancers [34]. Therefore, a high nitrate intake can increase the cancer risk. The cooking process modulates the nitrate contents in foods. The compounds formed when protein-rich foods and vegetables high in vitamins, minerals, antioxidants, and nitrates are fried can increase cancer risk. According to a study that performed a nitrate risk assessment of vegetables, the cooking process of raw vegetables reduced the nitrate content (4.094–59%), and the boiling process specifically decreased it the most (47–59%). However, the frying process of vegetables increased the nitrate content (12.46–29.93%) [34,35]. Reusing frying oil more than four times while cooking foods leads to a four-fold increased risk of digestive cancers [36]. The harmful components of frying oil can have different effects depending on the temperature and duration of cooking.

Although most studies found a significant increase in cancer risk with a high intake of fried foods (meat, fish, eggs, and potatoes), some studies found that the intake of heterocyclic amines within the usual dietary range from the frying process was unlikely to increase the incidence of cancer in the colon, rectum, bladder, or kidney [37]. In addition, it has been reported that the consumption of French fries was inversely proportional to the risk of pancreatic cancer [9]. Additionally, dietary acrylamide intake has not been associated with the risk of gastrointestinal cancer [38]. Furthermore, a lack of a significant association between fried/baked potato consumption and cancer risk has been reported [39]. As such, much of the epidemiological evidence is inconsistent.

Park et al. (2000) have shown that consuming French fries reduces gastric cancer. Previous association studies of pancreatic cancer and fried potatoes also suggested that the antioxidant properties of potatoes may have resulted in reducing the risk of pancreatic cancer [9]. Lee et al. (1995) have also reported a reduction in gastric cancer with the consumption of fried meat. These Korean studies were conducted about 30 years ago, when fat intake was less than 15 energy percent. Since eating habits have evolved significantly, these results should be interpreted cautiously. However, in one Korean study, donuts, a fried food, were found to increase the risk of *H. pylori* infection, which is one of the risk factors for GC [40]. *H. pylori* infection, dietary factors, and some culinary practices may act synergistically in the development of gastric cancer. Fried foods, including meats and bread, can potentially increase GC risk.

Epidemiologic changes have led to an increase in the incidence of gastric cancer in young people. Although the number of gastric cancer patients in the elderly group (48–85 years old) in Hehuang Valley, China, was three times that of the young group (18–45 years old), nearly 25% of cancer patients were from the young group [41]. A trend of higher gastric cancer among youth may be occurring worldwide, not just in China’s Hehuang Valley. This increase may be linked to an increased high-fat food intake, including fried foods, among the young compared to older people. A study in Algeria, North Africa, reported an 8.2-fold increased risk of gastric cancer when salted butter had been consumed for 20 years [36].

Frequent mealtime shifts over a long period increase the risk of *H. pylori* infection and gastritis [42]. A quantitative analysis of the relationship between irregular eating habits and gastric cancer in China showed that both irregular and fast eating were independent risk factors for gastric cancer (irregular eating: OR = 1.71, 95% CI: 1.62–1.91; fast eating: OR = 1.83; 95% CI: 1.71–2.01) [43]. It is common in Korea to enjoy eating fried chicken with beer as a late-night snack, and such habits could add to the risk of gastric cancer.

This meta-analysis has several limitations. (1) The case–control studies may have some bias, including selection, recall, and measurement, and the results should not be interpreted as causal. (2) Self-reported questionnaires may inevitably lead to some errors by participants regarding exposure. (3) The definition of the upper and lower categories of fried food intake was somewhat different in each study. (4) Some studies did not adjust for confounding factors, and the factors adjusted in each study were inconsistent. (5) Most studies were conducted before 2015, especially in non-East Asians, and this may indicate publication bias. However, the fried food intake was positively associated with the gastric cancer risk in meta-analyses of publications before and after 2015 in all studies with East Asian participants (Supplementary Figure S1A,B).

In conclusion, our meta-analysis, despite its limitations, provides compelling evidence of a positive correlation between fried food intake and the risk of gastric cancer. This association was observed in both non-East Asians and East Asians. The presence of potentially carcinogenic components in fried foods highlights the importance of considering the gastric cancer risk. Although the influence of oil types on the generation of carcinogenic components during the frying process was not extensively addressed in the selected studies, preliminary evidence suggests that olive oil may contain fewer carcinogenic components compared to other vegetable oils high in polyunsaturated fatty acids. However, this area requires further investigation. Given the practical difficulty of completely eliminating fried foods from diets, we recommend reducing the number of servings or limiting the amount per serving. Additionally, avoiding frying and cooking at excessively high temperatures is advisable. Future research should focus on prospective studies with larger sample sizes to establish a stronger correlation between fried food intake and gastric cancer risk. These studies would provide additional evidence and enhance our understanding of the relationship between fried food consumption and gastric cancer.

## Figures and Tables

**Table 1:** Summaries of the selected case–control studies.

First Author, (Year), Country Ref. No	Region, Study Design	Study Period (Years)	Population, Age	Inclusion Criteria (Exclusion Criteria Indicated)	Dietary Assessment
Demirer et al. (1990) Turkey [17]	Ankara, Turkey Case–control (Hospital-based)	1987–1988 (0.3)	100 cases and 100 controls (matched age, sex, patients; 29–78, healthy; 28–73)	Histologically proven adenocarcinoma of gastric cancer (cases) and same hospitals with no cancer or gastrointestinal disease (controls)	Frequency of consumption of specific food items past 15 years
Jedrychowski et al. (1992) Poland [18]	Poland Case–control (Hospital-based)	1986–1990 (5)	741 cases (histologically confirmed adenocarcinoma) and 741 controls (patients admitted to the surgical ward) <75 years	Not stated	Structured questionnaire
Lee et al. (1995) Korea [11]	Seoul, Korea Case–control (Hospital-based)	1990–1991 (1.3)	213 cases (histologically confirmed) and 213 matched (age, sex) controls 25–64 years, ≥65 years	Not diagnosed within 6 months preceding interview, aged >70, with other coexisting chronic systemic diseases affecting dietary patterns or communication problems, non-adenocarcinoma, recurrent cancers (cases), and hospitalized patients (controls)	Semiquantitative food frequency questionnaire with 64 food items, 3 years prior to the interview
Ji et al. (1998) China [19]	Shanghai, China Case–control (Population-based)	1988–1989 (1)	1124 cases and 1451 matched (age, sex) controls (randomly selected among Shanghai residents) 20–69 years	Not stated	Comprehensive food frequency questionnaire, 74 items assessing usual food intake 10 years before diagnosis
Sun et al. (1999) China [20]	Haerbin, China Case–control (Hospital-based)	1990–1996 (6.11)	360 cases and 360 healthy controls Average age: 57.94 years and 57.44 years	Not stated	Interview survey
Park et al. (2000) Korea [14]	Chungcheong province, Korea Case–control (Hospital-based)	Not stated	109 cases and 211 controls (matched age and sex)	Same hospitals without cancer	Direct interview with a structured questionnaire
De Stefani et al. (2001) Uruguay [23]	Montevideo, Uruguay Case–control (Hospital-based)	1997–1999 (2)	123 cases and 282 controls (patients with nonneoplastic diseases) 30–89 years	All newly diagnosed and histologically verified (cases) and same hospitals as the cases, not excessively ill patients (controls)	FFQ 64 items assessing intake 5 years before the onset of symptoms or interview
KoGES (2004–2013) Korea [28]	City-Cohort Korea Case–control (Hospital-based)	2004–2013 (9)	*n* = 48,306 312 cases and 47,994 controls ≥40 years	Without any history of cancer or disease of the digestive system	Semiquantitative food frequency questionnaire (SQFFQ) 106 items
Campos et al. (2006) Colombia [12]	Cali, Colombia Case–control (Hospital-based)	2000–2002 (2)	216 cases and 431 controls (noncancer patients matching in age, gender, and hospital) 49–75 years	Without recurrent GC, lived in Valle del Cauca for fewer than five years (cases) and malignant diseases, gastric illnesses, lived in Valle del Cauca for fewer than 5 years, severe clinical conditions, diagnosed with GC 15 years before (controls)	FFQ
Pakseresht, et al. (2011) Iran [26]	North-west Iran resided in Ardabil for over 20 years Case–control (Population-based)	2005–2007 (2)	286 cases and 304 noncancer controls ≥40 years	Without other malignancies (case)	QFFQ 117 items, assessing usual diet over the last year or 1 year before the diagnosis
Wang et al. (2012) China [16]	Jiangsu Province China Case–control (Population-based)	2004 (1)	249 cases and 260 healthy controls of the same geographic origins 57.6 ± 9.2 years	Without a history of other cancers or chronic diseases caused by dietary intake or communication problems (cases)	Semiquantitative food frequency table, last 10 years
Jiang et al. (2012) China [22]	Xian, China Permanent Resident (≥20 years) Case–control (Hospital-based)	2007–2010 (2.6)	217 cases and 245 healthy controls <45 years 45–64 years ≥65 years	Not stated	Epidemiological questionnaire
Sun et al. (2013) China [15]	Rural Areas of Linzhou, China Case–control (Population-based)	2005–2007 (2)	470 cases (newly diagnosed) and 470 healthy controls (matched age, sex) 40–69 years	Without any disease of the digestive system, living in the nearby Linzhou for more than 10 years (case)	Face-to-face interviews with a uniform questionnaire one year before the diagnosis
Somi et al. (2015) Iran [27]	East Azerbaijan of Iran Case–control (Hospital-based)	2009–2011 (2)	212 cases (21–84 years, Residents of East Azarbaijan for more than 20 years), 404 controls (cancer-free patients) 61.17 ± 11.72 years	Without other malignancies and a history of cancer, family history of cancer, and other gastrointestinal diseases (case)	Structured questionnaire, over the past two decades
Guo et al. (2018) China [21]	Henan Province, China Case–control (Population-based)	2005–2013 (8)	Endoscopic screening objects (*n* = 82,367) 331 cases and 6707 matched healthy controls 40–69 years Average age: (53.46 ± 8.07)	Inclusion: participating in the upper gastrointestinal cancer screening project for the first time; Exclusion: participants with missing pathological or endoscopic diagnosis information	Questionnaire
Cai et al. (2019) China [13]	China Cohort Case–control (Hospital-based)	2015–2017 (1.8)	Individuals with a high risk prior to gastroscopy (*n* = 14,929) 267 cases and 9571 controls 40–80 years	Exclusion: previous gastric operations, *H. pylori* eradication, taking H_2_ blockers or proton pump inhibitors in the past 2 weeks, a disorder of renal function, pregnancy, a history of any type of cancer, and a gastroscopy examination within 1 year	Pre-validated self-reported questionnaire
Huang et al. (2020) China [24]	Anhui, China Case–control (Hospital-based)	2016–2018 (2)	302 cases and 302 matched healthy controls Average 60 ± 11 and 59 ± 11 years	Without other malignancies, benign gastric diseases, dementia, severe dysfunction of organs, severe systematic unfitness, or first-degree relative having gastric cancer (cases)	Frequency score (FS)
Li et al. (2022) China [25]	Wannan, China Cohort Case–control (Hospital-based)	2019–2021 (2.5)	Prospectively enrolled asymptomatic participants (*n* = 25,194) 109 cases and 910 controls ≥40 years	Exclusion: taking H_2_ blockers or proton pump inhibitors in the past 2 weeks, *H. pylori* eradication, pregnant or lactating, chemotherapy or radiotherapy, mental symptoms, or other severe systemic diseases	Questionnaire survey

**Table 2:** Effects of fried foods on gastric cancer, as reported in the selected studies.

First Author, (Year), Country Ref. No	Sex	Types of Fried Foods or Cooking Methods	Fried Foods: High and Low Case/Control	Types of Gastric Cancer	Comparison (High vs. Low)	Adjusted OR, RR (95% CI)	Adjusted Covariates	Author’s Conclusion
Demirer et al. (1990) Turkey [17]	M/F	Fried (potatoes, meat, fish)	High: 8 (100)/ 5 (100) Low: 92 (100)/ 95 (100)	GC	Three to four times a week; Daily vs. No consumption; Rare consumption; Once or twice a month; Once or twice a week	not significant	Age, sex, blood group, Residential area	No difference in fried food consumption between the cases and controls
Jedrychowski et al. (1992) Poland [18]	M/F	Fried meat (usually served at home)	High: 141 (741)/ 105 (741) Low: 600 (741)/ 636 (741)	GC	Fried vs. No	RR 2.06 (1.48–2.87)	Age, sex, education, occupation of the index person, and residency	Fried or stewed food was associated with a significantly higher risk for GC compared to boiling
Lee et al. (1995) Korea [11]	M/F	Fried (meat, fish)	High: 30 (213)/ 56 (213) Low: 183 (213)/ 157 (213)	GC	High, vs. Intermediate Low	0.4 (0 2–0.8) 0.8 (0.5–1.3) 1.0 (*p* < 0.001)	Age, sex, education, economic status, and residence areas	Frying (fried meat, fish) was associated with a decreased GC risk
Ji et al. (1998) China [19]	M/F	Fried food	High: 131 (1122)/88 (1446) Low: 991 (1122)/ 1358 (1446)	Overall GC (Cardia, Distal)	Frequently Sometimes vs. Occasionally	2.3 (1.6–3.2) 1.1 (0.9–1.4) 1.0 (*p* = 0.0001)	Age, sex, income, education, smoking, and alcohol drinking	Risks for both cardia and non-cardia tumors increased by 2-fold with frequent consumption of fried foods
Sun et al. (1999) China [20]	M/F	Fried food	High: 67 (360)/ 20 (360) Low: 293 (360)/ 340 (360)	GC	Frequently vs. Occasionally; Do not eat; Rarely eat	1.20 (0.67–2.16) 3.02 (1.41–6.47) 1.00 1.31 (0.73–1.77)	Age, sex, education, and others	Regular fried food consumption can increase the RR of gastric cancer by 3 times. Regularly consuming garlic, vinegar, and soy products can reduce the GC risk.
Park et al. (2000) Korea [14]	M/F	Fried potatoes	High: 6 (109)/ 2 (211) Low: 99 (109)/ 205 (211)	GC	4 times or more per month, vs. Less than twice per month, 2 or 3 times per month, or 2 or 3 times per week,	*p* = 0.019	Age, sex, smoking, drinking, BMI	Eating more fried potatoes was associated with a lower risk of GC
De Stefani et al. (2001) Uruguay [23]	M/F	Frying cooking method	High: 17 (123)/ 44 (264) Low: 106 (123)/ 220 (264)	Overall GC	Fried vs. No	0.9 (0.4–1.8)	Age, gender, education, residence, urban/rural status, smoking, alcohol, mate drinking, energy, and meat intake.	No association
KoGES. (2004–2013) Korea [28]	M/F	Fried foods	High: 11 (312)/ 2481 (47,994) Low: 300 (312)/ 45,184 (47,994)	GC	≥1 time/week vs. <1 time/week or Never	0.933 (0.493–1.766) 1.0	Age, gender, BMI, residence area, physical activity, education, income, smoking, intake of alcohol and energy	Not significance
Campos et al. (2006) Colombia [12]	M/F	Fried foods	High: 203 (216)/ 382 (431) Low: 13 (216)/ 49 (431)	GC	Yes vs. No	1.9 (1.0–3.6) (*p* = 0.039)	Age, gender	A significant association between GC risk and fried foods
Pakseresht et al. (2011) Iran [26]	M/F	Fried foods	High: 132 (286)/ 116 (304) Low: 154 (286)/ 188 (304)	Total GC Cardia Non-cardia Intestinal Diffuse	Yes vs. No	2.21 (1.45–3.37) 4.91 (2.19–11.06) 2.17 (1.34–3.50) 3.33 (1.90–5.82) 3.96 (1.83–8.56)	Age, sex, education, income, living area, smoking, total energy intake, gastric symptoms, *H. pylori* infection, owning a refrigerator, period of using a refrigerator, frying method	Positive associations with people who prefer fried foods
Wang D et al. (2012) China [16]	M/F	Fried foods (beef, pork, lamb, fish, egg, vegetables)	High: 138 (249)/ 96 (260) Low: 111 (249)/ 164 (260)	Genotypes of GC IVS10 + 12G > A IVS12-6T > C	≥2.5 portions per week vs. less	2.88 (1.70–4.94) (*p* < 0.001) 2.48 (1.42–4.13) (*p* = 0.007)	Age, gender, smoking, drinking, and pickled food intake	High intake of fried foods was positively correlated among participants, particularly with the IVS12-6T > C or IVS10 + 12G > A
Jiang M et al. (2012) China [22]	M/F	Fried foods	High: 174 (217)/ 176 (245) Low: 43 (217)/ 69 (245)	GC	≥3 times/week <3 times/week	4.372 (1.633–11.706) *p* = 0.003	Age, sex, level of education, family history of GC or HP infection	Barbecued food, fried food, overheating diet, and salty food were risk factors for GC, while green tea and garlic intake were protective factors.
Sun et al. (2013) China [15]	M/F	Fried foods	High: 144 (470)/ 113 (470) Low: 326 (470)/ 357 (470)	Cardia GC	≥2 times/week 1–2 times/week Never	1.46 (0.91–2.33) (*p* = 0.038) 1.06 (0.69–1.63) (*p* = 0.578) 1	Age, sex, nationality, marital status, per capita income, education level	Fried food intake was a risk factor for GC cancer
Somi et al. (2015) Iran [27]	M/F	Deep-fried meat (Ghorme), high-fat food	High: 58 (212)/ 64 (404) Low: 145 (212)/ 315 (404)	GC	Yes vs. No	2.47 (1.5–4.07) (*p* < 0.001)	Sex, age, education, BMI, smoking	There were positive associations between high-fat foods and GC risk.
Guo et al. (2018) China [21]	M/F	Fried food	High: 34 (331)/ 708 (6707) Low: 297 (331)/ 5999 (6707)	GC	Frequently (≥2 times/week), occasionally (1~2 times/week) do not eat (<1 time/week),	1.91 (1.66–2.20) (*p* < 0.001) 1.89 (1.57–2.28) (*p* < 0.001) 1.00	Age, gender, BMI, marital status, education, smoking, intake of alcohol	Eating fried food is a risk factor for GC and precancerous lesions. Reducing the intake of fried food can prevent the occurrence of gastric carcinoma and precancerous lesions.
Cai et al. (2019) China [13]	M/F	Fried foods	High: 36 (267)/ 672 (9571) Low: 231 (267)/ 8899 (9571)	GC	Regular (at least three times/week) vs. occasional (<3 times/week)	1.71 (1.15–2.54) (*p* = 0.008)	Age, sex, anti-*H. pylori*, PG I/II ratio, IgG status, G-17 concentration, pickled food, fried food	Fried food consumption is a predictor of gastric cancer
Huang et al. (2020) China [24]	M/F	Fried foods	High: 41 (302)/ 33 (302) Low: 264 (302)/ 269 (302)	GC	Food frequency scores: 1 score ≤1 time per month; 2 scores 2–3 times per month; 0 scores Never	1.07 (0.95–1.21) (*p* = 0.265)	Age, sex, height, weight, education level, marital status, alcohol drinking, smoking, and passive smoking	The habit of cooking fried food was associated with higher GC incidence
Li et al. (2022) China [25]	M/F	Fried foods	High: 23 (109)/ 94 (910) Low: 86 (109)/ 816 (910)	GC and precancerous lesions	Yes vs. No	2.322 (1.398–3.855) (*p* = 0.001)	GC, *H. pylori* infection	Frequent consumption of fried foods was an independent risk factor for GC and precancerous lesions

ORs, odds ratio; RR, relative risk; GC, gastric cancer.

## Data Availability

Data will be available upon request with reasonable reasons.

## Supplementary Materials

The following supporting information can be downloaded at: https://www.mdpi.com/article/10.3390/nu15132982/s1, Table S1. Quality assessment of case–control studies (*n* = 18). Figure S1. Forest plot of the association between high fried food intake and gastric cancer risk according to publication date.
