# Supplementary material for: Association of Fried Food Intake with Gastric Cancer Risk: A Systemic Review and Meta-Analysis of Case–Control Studies
Source: Nutrients. 2023 Jun 30;15(13):2982. doi: 10.3390/nu15132982 (PMC10347084; doi:10.3390/nu15132982)
Supplement: Supplementary file 1 [file nutrients-15-02982-s001.zip › nutrients-2452819-Figure S1.pdf]

Supplementary Figure S1. Forest plot of the association between high fried food intake and gastric cancer risk according to publication date.

A. Total participants according to before and after 2015

Study or Subgroup	GC Events	GC Total	Non-GC Events	Non-GC Total	Weight	Odds Ratio M-H, Random, 95% CI	Year
**2.3.1 ≤ 2015**							
Demirer T et al. 1990	8	100	5	100	2.4%	1.65 [0.52, 5.24]	1990
Jedrychowskii W et al. 1992	141	741	105	741	7.1%	1.42 [1.08, 1.88]	1992
Lee JK et al. 1995	30	213	56	213	5.6%	0.46 [0.28, 0.75]	1995
Ji BT et al. 1998	131	1122	88	1446	7.0%	2.04 [1.54, 2.71]	1998
Sun XW et al. 1999	67	360	20	360	5.4%	3.89 [2.30, 6.56]	1999
Park JS et al. 2000	6	109	2	211	1.4%	6.09 [1.21, 30.69]	2000
De Stefani E et al. 2001	17	123	44	264	4.9%	0.80 [0.44, 1.47]	2001
Campos F et al. 2006	203	216	382	431	4.7%	2.00 [1.06, 3.78]	2006
Pakseresht M et al. 2011	132	286	116	304	6.7%	1.39 [1.00, 1.93]	2011
Wang D et al. 2012	138	249	96	260	6.6%	2.12 [1.49, 3.03]	2012
Jiang M et al. 2012	174	217	176	245	6.0%	1.59 [1.03, 2.45]	2012
Sun CQ et al. 2013	144	470	113	470	7.0%	1.40 [1.05, 1.86]	2013
KoGES. 2004-2013	11	312	2481	47994	4.9%	0.67 [0.37, 1.23]	2013
Somi MH et al. 2015	58	212	64	404	6.2%	2.00 [1.34, 2.99]	2015
**Subtotal (95% CI)**		**4730**		**53443**	**75.8%**	**1.51 [1.17, 1.94]**	
Total events	1260		3748				
Heterogeneity: Tau² = 0.17; Chi² = 60.18, df = 13 (P < 0.00001); I² = 78%							
Test for overall effect: Z = 3.15 (P = 0.002)							
**2.3.2 > 2015**							
Guo LW et al.2018	34	331	708	6707	6.5%	0.97 [0.67, 1.39]	2018
Cai Q et al. 2019	36	267	672	9571	6.5%	2.06 [1.44, 2.96]	2019
Huang L et al. 2020	41	302	33	302	5.6%	1.28 [0.79, 2.09]	2020
Li L et al. 2022	23	109	94	910	5.5%	2.32 [1.40, 3.86]	2022
**Subtotal (95% CI)**		**1009**		**17490**	**24.2%**	**1.55 [1.01, 2.36]**	
Total events	134		1507				
Heterogeneity: Tau² = 0.14; Chi² = 11.97, df = 3 (P = 0.007); I² = 75%							
Test for overall effect: Z = 2.03 (P = 0.04)							
**Total (95% CI)**		**5739**		**70933**	**100.0%**	**1.52 [1.23, 1.87]**	
Total events	1394		5255				
Heterogeneity: Tau² = 0.15; Chi² = 72.05, df = 17 (P < 0.00001); I² = 76%							
Test for overall effect: Z = 3.84 (P = 0.0001)							
Test for subgroup differences: Chi² = 0.01, df = 1 (P = 0.91), I² = 0%							

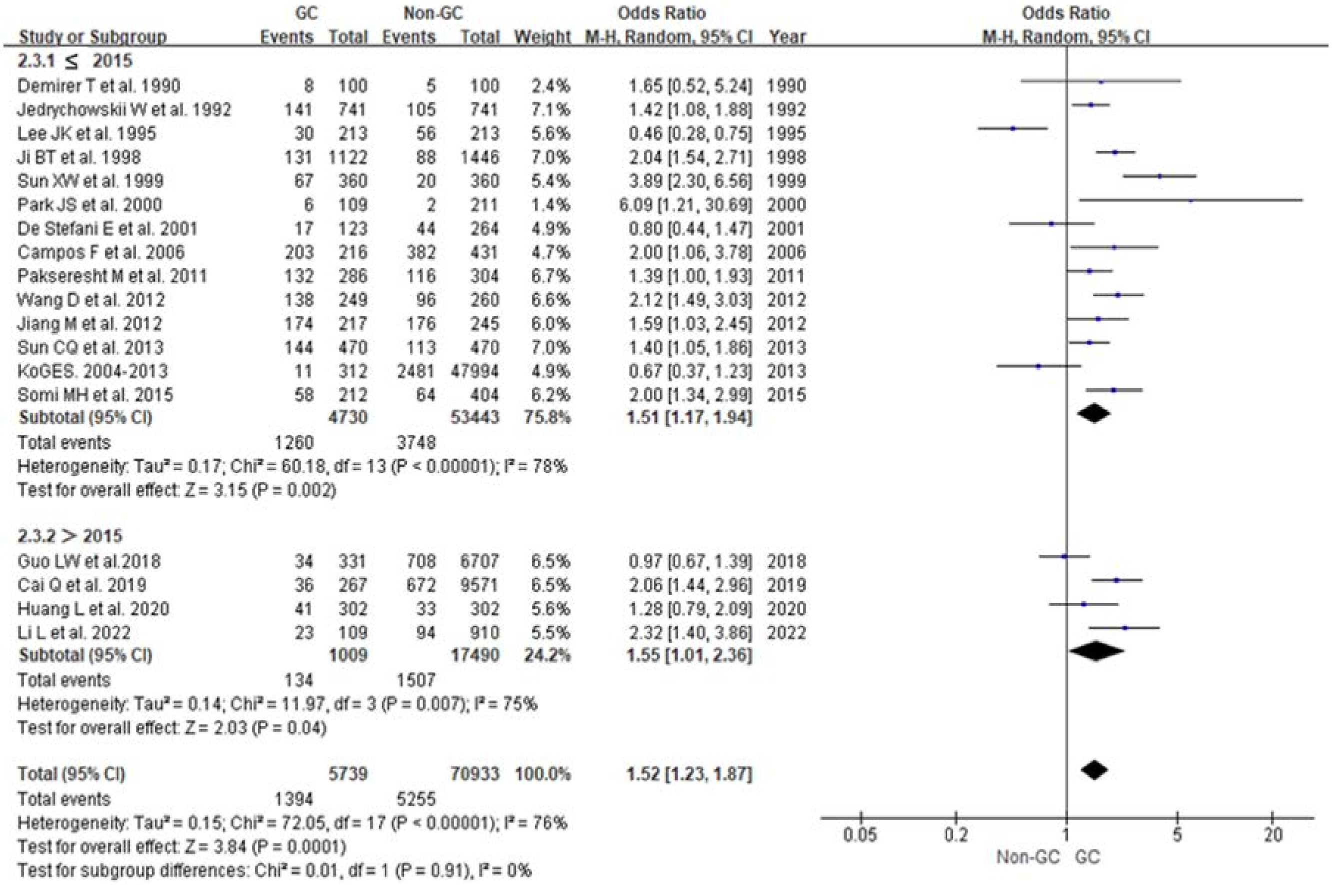

B. East Asians according to before and after 2015

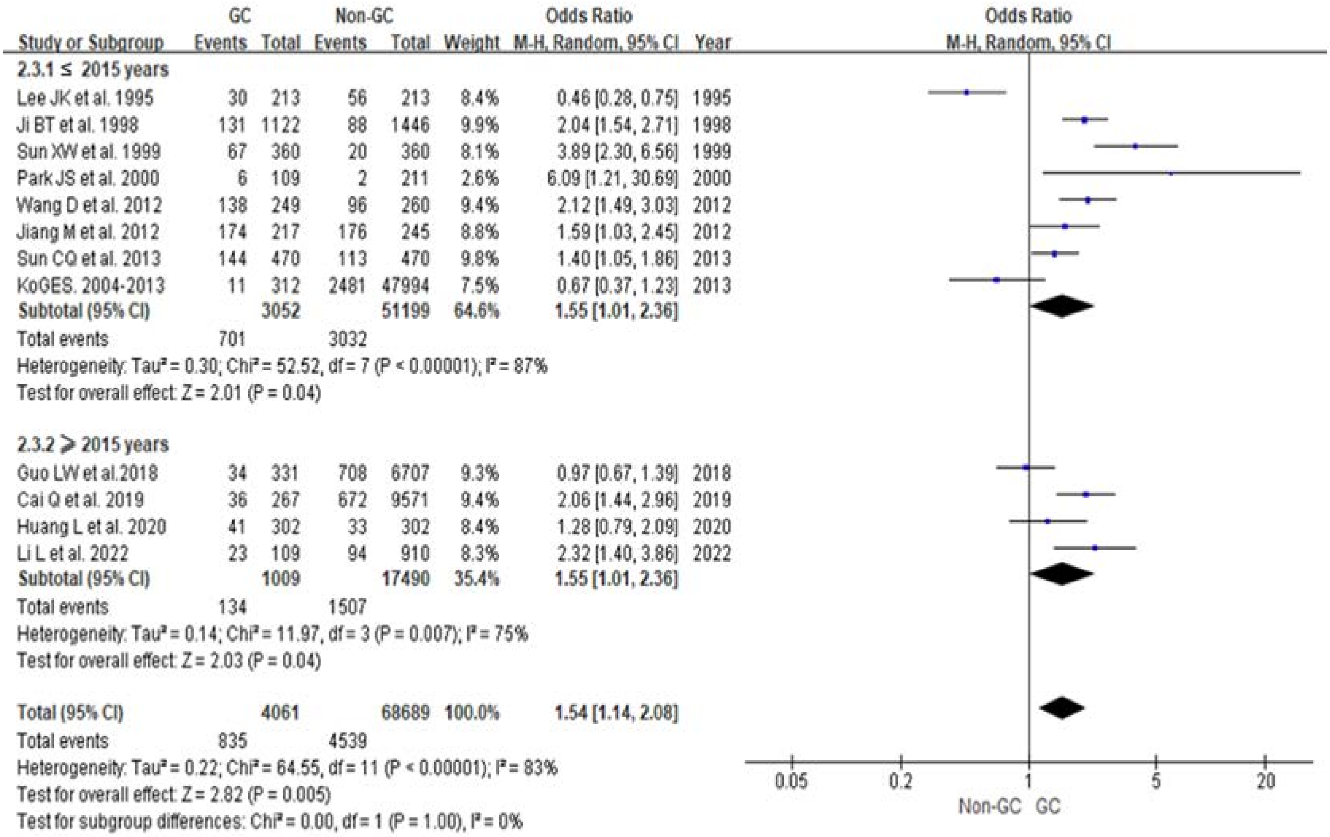

Study or Subgroup	GC Events	GC Total	Non-GC Events	Non-GC Total	Weight	Odds Ratio M-H, Random, 95% CI	Year
**2.3.1 ≤ 2015 years**							
Lee JK et al. 1995	30	213	56	213	8.4%	0.46 [0.28, 0.75]	1995
Ji BT et al. 1998	131	1122	88	1446	9.9%	2.04 [1.54, 2.71]	1998
Sun XW et al. 1999	67	360	20	360	8.1%	3.89 [2.30, 6.56]	1999
Park JS et al. 2000	6	109	2	211	2.6%	6.09 [1.21, 30.69]	2000
Wang D et al. 2012	138	249	96	260	9.4%	2.12 [1.49, 3.03]	2012
Jiang M et al. 2012	174	217	176	245	8.8%	1.59 [1.03, 2.45]	2012
Sun CQ et al. 2013	144	470	113	470	9.8%	1.40 [1.05, 1.86]	2013
KoGES. 2004-2013	11	312	2481	47994	7.5%	0.67 [0.37, 1.23]	2013
**Subtotal (95% CI)**		**3052**		**51199**	**64.6%**	**1.55 [1.01, 2.36]**	
Total events	701		3032				
Heterogeneity: Tau² = 0.30; Chi² = 52.52, df = 7 (P < 0.00001); I² = 87%							
Test for overall effect: Z = 2.01 (P = 0.04)							
**2.3.2 > 2015 years**							
Guo LW et al.2018	34	331	708	6707	9.3%	0.97 [0.67, 1.39]	2018
Cai Q et al. 2019	36	267	672	9571	9.4%	2.06 [1.44, 2.96]	2019
Huang L et al. 2020	41	302	33	302	8.4%	1.28 [0.79, 2.09]	2020
Li L et al. 2022	23	109	94	910	8.3%	2.32 [1.40, 3.86]	2022
**Subtotal (95% CI)**		**1009**		**17490**	**35.4%**	**1.55 [1.01, 2.36]**	
Total events	134		1507				
Heterogeneity: Tau² = 0.14; Chi² = 11.97, df = 3 (P = 0.007); I² = 75%							
Test for overall effect: Z = 2.03 (P = 0.04)							
**Total (95% CI)**		**4061**		**68689**	**100.0%**	**1.54 [1.14, 2.08]**	
Total events	835		4539				
Heterogeneity: Tau² = 0.22; Chi² = 64.55, df = 11 (P < 0.00001); I² = 83%							
Test for overall effect: Z = 2.82 (P = 0.005)							
Test for subgroup differences: Chi² = 0.00, df = 1 (P = 1.00), I² = 0%							
