# Supplementary material for: Association of Fried Food Intake with Gastric Cancer Risk: A Systemic Review and Meta-Analysis of Case–Control Studies
Source: Nutrients. 2023 Jun 30;15(13):2982. doi: 10.3390/nu15132982 (PMC10347084; doi:10.3390/nu15132982)
Supplement: Supplementary file 1 [file nutrients-15-02982-s001.zip › nutrients-2452819-Table S1.pdf]

Supplementary Table S1: Quality assessment of case-control studies (n = 18)

First author, (Year), Country Ref. no	Research question	Study population	Target population and case representation	Sample size justification	Groups recruited from the same population	Inclusion and exclusion criteria prespecified and applied uniformly	Case and control definitions	Random selection of study participants	Concurrent controls	Exposure assessed prior to outcome measurement	Exposure measures and assessment	Blinding of exposure assessors	Statistical analysis	Tota score
Demirer et al. (1990) Turkey [17]	0	0	0	2	0	2	0	1	2	0	1	2	2	[illegible]
Jedrychowski et al. (1992) Poland [18]	0	0	0	2	0	2	0	1	2	1	1	2	0	[illegible]
Lee et al. (1995) Korea [11]	0	0	0	0	0	0	0	1	2	0	1	2	0	[illegible]
Ji et al. (1998) China [19]	0	0	0	0	0	2	0	0	2	0	1	2	0	[illegible]
Sun et al. (1999) China [20]	0	0	0	0	2	2	0	0	1	2	1	2	0	[illegible]
Park et al. (2000) Korea [14]	0	0	0	2	0	2	0	1	2	1	1	2	2	[illegible]
De Stefani et al. (2001) Uruguay [23]	0	0	0	0	0	2	0	1	2	0	1	2	0	[illegible]
KoGES 2004-2013 Korea [28]	0	0	0	2	0	0	0	1	2	0	1	0	0	[illegible]
Campos et al. (2006) Colombia [12]	0	0	0	0	0	0	0	1	1	1	1	1	2	[illegible]
Pakseresht et al.	0	0	0	2	2	2	0	0	1	0	1	2	0	[illegible]

(2011) Iran [26]													
Wang et al. (2012) China [16]	0	0	0	2	0	0	0	1	1	0	1	2	0
Jiang et al. (2012) China [22]	0	0	0	0	2	2	0	1	1	1	2	2	1
Sun et al. (2013) China [15]	0	0	0	2	0	0	0	0	0	0	1	2	2
Somi et al. (2015) Iran [27]	0	0	0	2	0	0	0	1	1	0	1	2	0
Guo et al. (2018) China [21]	0	0	0	0	0	0	0	1	2	1	1	1	0
Cai Q et al. (2019) China [13]	0	0	0	0	0	0	0	1	0	0	1	0	0
Huang et al. (2020) China [24]	0	0	0	2	0	0	0	1	1	2	1	1	0
Li et al. (2022) China [25]	0	0	0	0	0	0	0	1	0	0	1	1	0

Note: 0, yes; 1, not applicable or not reported; 2, no.
